# Immune inhibitory function of bovine CTLA-4 and the effects of its blockade in IFN-γ production

**DOI:** 10.1186/s12917-019-2082-7

**Published:** 2019-10-29

**Authors:** Kei Watari, Satoru Konnai, Naoya Maekawa, Tomohiro Okagawa, Yasuhiko Suzuki, Shiro Murata, Kazuhiko Ohashi

**Affiliations:** 1 Department of Disease Control, Faculty of Veterinary Medicine, Hokkaido University, Sapporo, Hokkaido 060-0818 Japan; 2 Department of Advanced Pharmaceutics, Faculty of Veterinary Medicine, Hokkaido University, Sapporo, 060-0818 Japan; 3 Division of Bioresources, Research Center for Zoonosis Control, Hokkaido University, Sapporo, 001-0020 Japan; 4 Global Station for Zoonosis Control, Global Institution for Collaborative Research and Education (GI-CoRE), Hokkaido University, Sapporo, 001-0020 Japan

**Keywords:** Cattle, CTLA-4, CD80, CD86, IFN-γ, BLV

## Abstract

**Background:**

Cytotoxic T-lymphocyte antigen 4 (CTLA-4) is known as an immune inhibitory receptor that is expressed on activated effector T cells and regulatory T cells. When CTLA-4 binds to CD80 or CD86, immunoinhibitory signals are transmitted to retain a homeostasis of the immune response. Recent studies have reported that CTLA-4 is upregulated in chronic infections and malignant neoplasms, contributing to host immune dysfunction. On the other hand, the blockade of CTLA-4 and CD80 or CD86 binding by antibody restores the immune response against these diseases. In a previous report, we indicated that the expression of CTLA-4 was closely associated with disease progression in cattle infected with the bovine leukemia virus (BLV). In this study, we established an anti-bovine CTLA-4 antibody to confirm its immune enhancing effect.

**Results:**

Bovine CTLA-4-Ig binds to bovine CD80 and CD86 expressing cells. Additionally, CD80 and CD86 bind to CTLA-4 expressing cells in an expression-dependent manner. Bovine CTLA-4-Ig significantly inhibited interferon-gamma (IFN-γ) production from bovine peripheral blood mononuclear cells (PBMCs) activated by Staphylococcus enterotoxin B (SEB). An established specific monoclonal antibody (mAb) for bovine CTLA-4 specifically recognized only with bovine CTLA-4, not CD28, and the antibody blocked the binding of CTLA-4-Ig to both CD80 and CD86 in a dose-dependent manner. The bovine CTLA-4 mAb significantly restored the inhibited IFN-γ production from the CTLA-4-Ig treated PBMCs. In addition, the CTLA-4 mAb significantly enhanced IFN-γ production from CTLA-4 expressing PBMCs activated by SEB. Finally, we examined whether a CTLA-4 blockade by CTLA-4 mAb could restore the immune reaction during chronic infection; the blockade assay was performed using PBMCs from BLV-infected cattle. The CTLA-4 blockade enhanced IFN-γ production from the PBMCs in response to BLV-antigens.

**Conclusions:**

Collectively, these results suggest that anti-bovine CTLA-4 antibody can reactivate lymphocyte functions and could be applied for a new therapy against refractory chronic diseases. Further investigation is required for future clinical applications.

## Background

The immune system is activated to eliminate viruses, bacteria, and tumor cells. This reaction is to protect against diseases and to maintain homeostasis. However, when the immune system is activated for an extended period, it could attack healthy tissue and cause an adverse effect. Cytotoxic T-Lymphocyte antigen-4 (CTLA-4), known as CD152, has an immunosuppressive role and suppresses an unrestrained immune response against self-antigen [1, 2]. CTLA-4 is expressed on regulatory T cells and activated effector T cells. CTLA-4 binds to CD80 (B7–1)/CD86 (B7–2) expressed on antigen-presenting cells and suppresses host immunity [3, 4].

CTLA-4 plays an essential role in the adjustment of the host immune system, but its expression is upregulated in several chronic infections and cancers. In all stages of a human immunodeficiency virus (HIV) infection, CTLA-4 expression is upregulated on CD4^+^ T cells and the virus load positively correlates with disease progression [5].

It has been reported that CTLA-4 expression on regulatory T cells increases with the disease progression of a HIV infection [6]. Patients infected with the hepatitis C virus (HCV) also have a higher level of CTLA-4 expression on liver CD8^+^ T cells [7]. The overexpression of CTLA-4 is associated with a worse prognosis in patients with nasopharyngeal carcinoma [8]. Thus, CTLA-4 contributes to the inhibition of the host immune system and exacerbates chronic infections and tumors.

Bovine leukemia virus (BLV) is a retrovirus that persistently infects B cells in cattle [9, 10] and causes stepwise disease progression. Most BLV-infected cattle have aleukemic leukemia, but 20–30% of BLV-infected cattle develop persistent lymphocytosis, which shows neoplasia of polyclonal B cells. Furthermore, 2–3% of BLV-infected cattle develop enzootic bovine leukemia and malignant lymphosarcoma will form in the lymphocytes resulting in death [9]. In previous studies, we elucidated the proportion of regulatory T cell increase in line with disease progression. As a result of the increased number of regulatory T cells, transforming growth factor beta (TGF-β) production upregulates and downregulates the expression of interferon-gamma (IFN-γ) and tumor necrosis factor (TNF-α) from CD4^+^ T cells which results in the suppression of natural killer (NK) cells [11, 12]. In addition, we also found that programmed cell death-1 (PD-1)/programmed cell death ligand-1 (PD-L1) and lymphocyte activation gene 3 (LAG-3) are related to BLV infection [13–15]. Furthermore, CTLA-4 expression has been reported to be upregulated because of the disease progression of the BLV infection [16]. Results from the same study show that the high expression of immune inhibitory molecules is related to disease progression of BLV infection. However, little is known about the CTLA-4 function in cattle.

In this study, we generated bovine CTLA-4-Ig, a recombinant bovine CTLA-4 fused with rabbit IgG, to confirm the immunoinhibitory function of bovine CTLA-4. Additionally, we generated a bovine CTLA-4 specific monoclonal antibody (mAb) by using CTLA-4-Ig with the ability to block the binding of CTLA-4 and CD80/CD86. Then we applied the bovine CTLA-4 mAb to examine the enhancement of IFN-γ production in BLV-infected cattle.

## Results

### Generation of recombinant bovine CTLA-4-Ig, CD80-Ig, and CD86-Ig

**Fig. 1:**
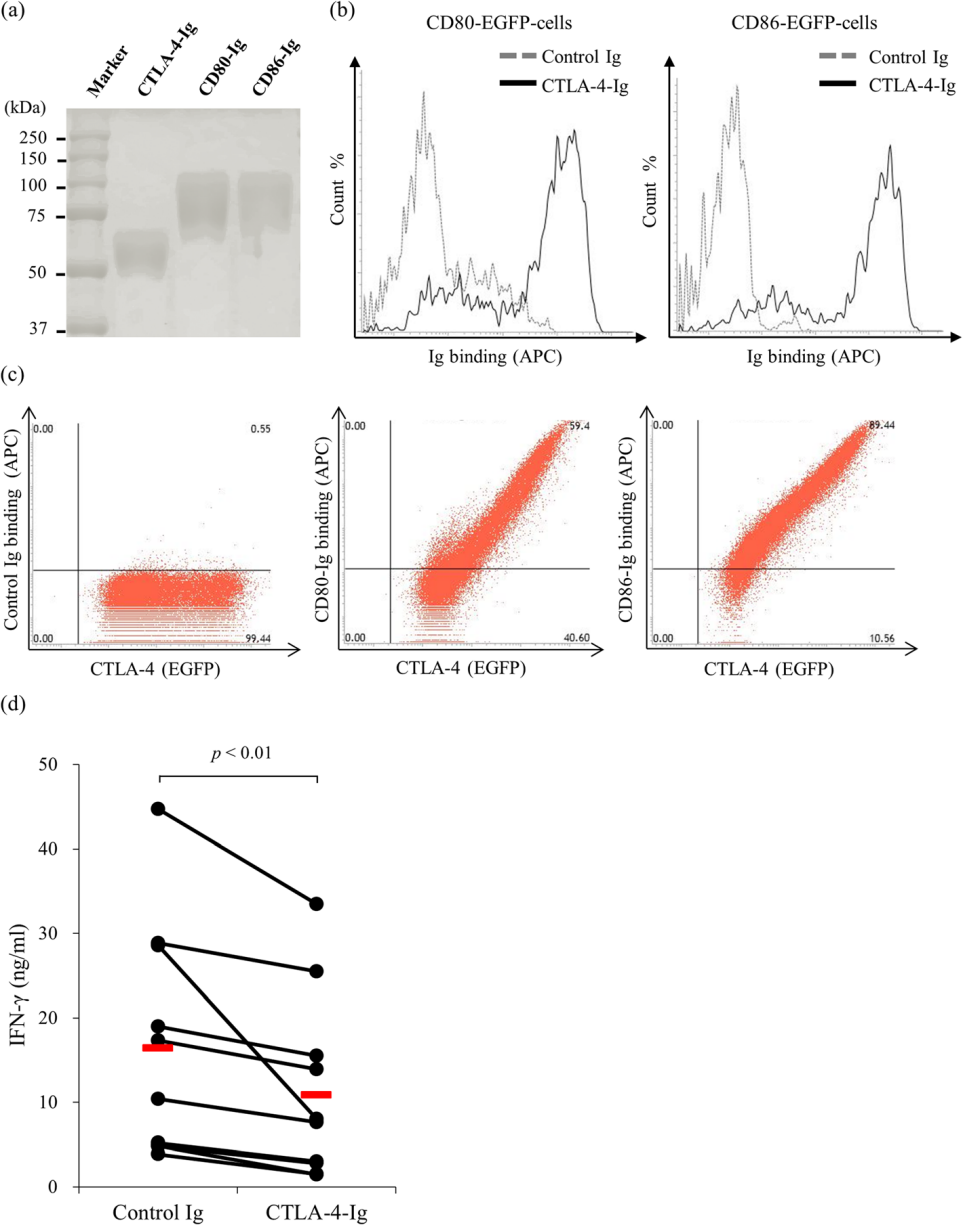
The generation of recombinant proteins and binding conformations of CD80/CD86-Ig and functional analysis of cytotoxic T-lymphocyte antigen 4 (CTLA-4)-Ig. CTLA-4-Ig, CD80-Ig, and CD86-Ig were produced in Expi293F cells that were transfected with expression vectors. **a** The purification of CTLA-4-Ig, CD80-Ig, and CD86-Ig was confirmed using SDS-PAGE. **b** The binding of CTLA-4-Ig against CD80-enhanced green fluorescent protein (EGFP) or CD86-EGFP expressing Cos-7 cells was confirmed using flow cytometry. **c** The binding of rabbit IgG, CD80-Ig, and CD86-Ig against CTLA-4-EGFP expressing CHO cells was confirmed using flow cytometry. **d** Peripheral blood mononuclear cells (PBMCs) (*n* = 11) were isolated from healthy cattle and cultured with 10 nM rabbit IgG or CTLA-4-Ig in the presence of Staphylococcus enterotoxin B (SEB). The cell culture supernatant was harvested after 7 days, and an ELISA was used to measure the inhibited interferon-gamma (IFN-γ) concentration. The bars indicate the average of each group. The statistical comparisons between each group were made using the Wilcoxon matched-pairs test. Differences were considered statistically significant at *p* < 0.01

In order to generate bovine CTLA-4-Ig, CD80-Ig, and CD86-Ig, we used the nucleotide sequences of CTLA-4, CD80, and CD86 that are registered in the NCBI nucleotide database. We used an analyzing tool to predict the signal peptide, extracellular region, transmembrane region, and intracellular region of each protein. Each protein’s signal peptide and extracellular region was inserted into a pCXN2.1-Rabbit IgG Fc vector, and expression plasmids were established. Then, the established plasmids were transfected to Expi293F cells and the expressed recombinant protein was confirmed by sodium dodecyl sulfate-polyacrylamide gel electrophoresis (SDS-PAGE). The molecular weight of CTLA-4-Ig, CD80-Ig, and CD86-Ig was 50, 70, and 70 kDa, respectively (Fig. 1a). Impurities were not found with SDS-PAGE, meaning that the purity of the established recombinant proteins was sufficient.

### Confirmation of bovine CTLA-4 binding to bovine CD80 and CD86 and its immune inhibitory function

The binding of bovine CTLA-4-Ig to bovine CD80 and CD86 was analyzed using FACS. We created CD80-enhanced green fluorescent protein (EGFP) and CD86-EGFP expressing Cos-7 cells to confirm the binding of CTLA-4-Ig against CD80 and CD86. As expected, CTLA-4-Ig strongly bound to CD80 and CD86 (Fig. 1b). In addition, we created CTLA-4-EGFP expressing CHO cells and verified the binding of CD80-Ig and CD86-Ig to bovine CTLA-4. CD80 and CD86 bind to CTLA-4 expressing cells in an expression-dependent manner, whereas the binding was not detected when using the control Ig (Fig. 1c). Both of these findings indicate that bovine CTLA-4 binds to its ligands, CD80 and CD86. In addition, we confirmed the immune inhibitory function of bovine CTLA-4. Peripheral blood mononuclear cells (PBMCs) were isolated from healthy cattle and cultured with CTLA-4-Ig or rabbit IgG in the presence of 0.1 μg/ml SEB. CTLA-4-Ig significantly inhibited the production of IFN-γ compared with the isotype control (Fig. 1d).

### Blockade of the CTLA-4 mAb in CTLA-4 and its ligand bindings

**Fig. 2:**
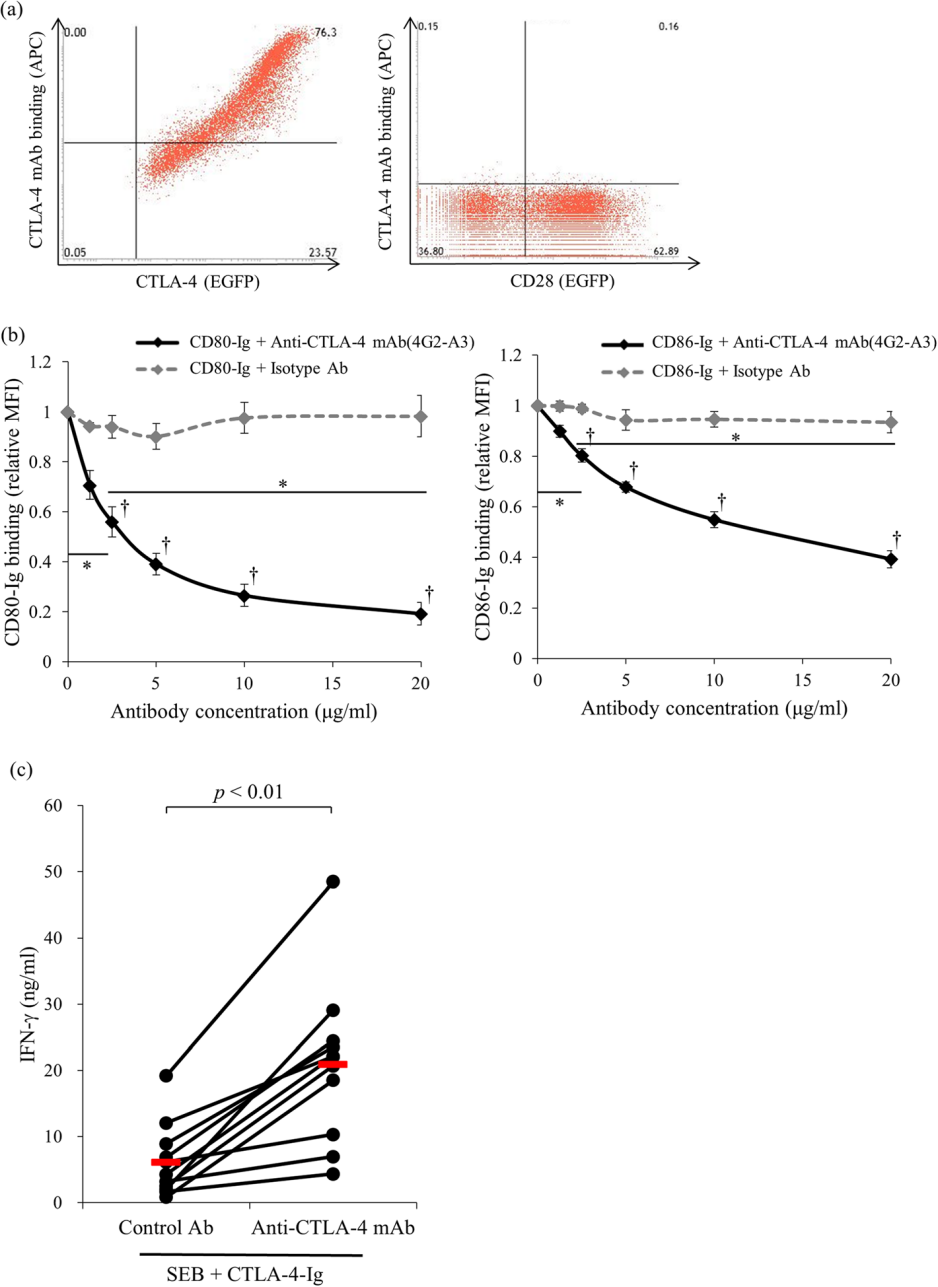
The binding and blocking ability of an established anti-CTLA-4 mAb. **a** The binding of anti-CTLA-4 mAb against CTLA-4-EGFP expressing CHO cells and CD28-EGFP expressing Cos-7 cells was measured using flow cytometry. **b** A dose-dependent blocking effect of 4G2-A3 on CTLA-4/CD80 and CTLA-4/CD86 binding. CTLA-4-EGFP cells were preincubated with 4G2-A3 or isotype Ab at various concentrations (1.25, 2.5, 5, 10, and 20 μg/mL), and the Ig binding was detected using flow cytometry. Each point indicates the average value of relative MFI obtained from three independent experiments (compared to no antibody control, error bar; SEM). Tukey’s test was used for the statistical analysis (* *p* < 0.05, between the group with 0 and 2.5 μg/ml of anti-CTLA-4 antibody and the group with 2.5 and 20 of anti-CTLA-4 antibody. † *p* < 0.05, between the anti-CTLA-4 antibody treated group and isotype antibody treated group of each concentration.). **c** PBMCs (*n* = 11) were isolated from healthy cattle and cultured with 20 μg/ml of control Ab or anti-CTLA-4 mAb in the presence of SEB and 10 nM CTLA-4-Ig. The culture medium was harvested after 7 days, and an ELISA was used to measure the IFN-γ concentration. The bars indicate the average of each group. Statistical comparisons between each group were made using the Wilcoxon matched-pairs test. Differences were considered statistically significant at *p* < 0.01

We established anti-bovine CTLA-4 mAb by immunizing CTLA-4-Ig to mice. Lymphocytes were harvested from the immunized mouse iliac lymph node and 600 hybridomas that produce anti-bovine CTLA-4 polyclonal antibodies were established. A flow cytometry analysis and ELISA confirmed the polyclonal antibody’s binding ability. Three clones were selected from 600 hybridomas and 300 clones of hybridomas that produce anti-bovine CTLA-4 monoclonal antibody were established using a methylcellulose method and limiting dilution method. From 300 clones of hybridoma, a 4G2-A3 clone was selected and applied in this study. The purified antibody bound explicitly to bovine CTLA-4 expressing cells in an expression-dependent manner. Meanwhile, the antibody did not bind to bovine CD28 expressing Cos-7 cells (Fig. 2a). Interestingly, the antibody actively blocked the binding of CTLA-4-Ig to both CD80 and CD86 in a dose-dependent manner (Fig. 2b). Thus, we confirmed whether the antibody could restore IFN-γ production from CTLA-4-Ig treated cells (Fig. 1d) by CTLA-4 blockade. Interestingly, the bovine CTLA-4 mAb significantly restored the inhibited IFN-γ production from the CTLA-4-Ig treated PBMCs, while the control Ig did not (Fig. 2c).

### The increase of IFN-γ production by CTLA-4 blockade

**Fig. 3:**
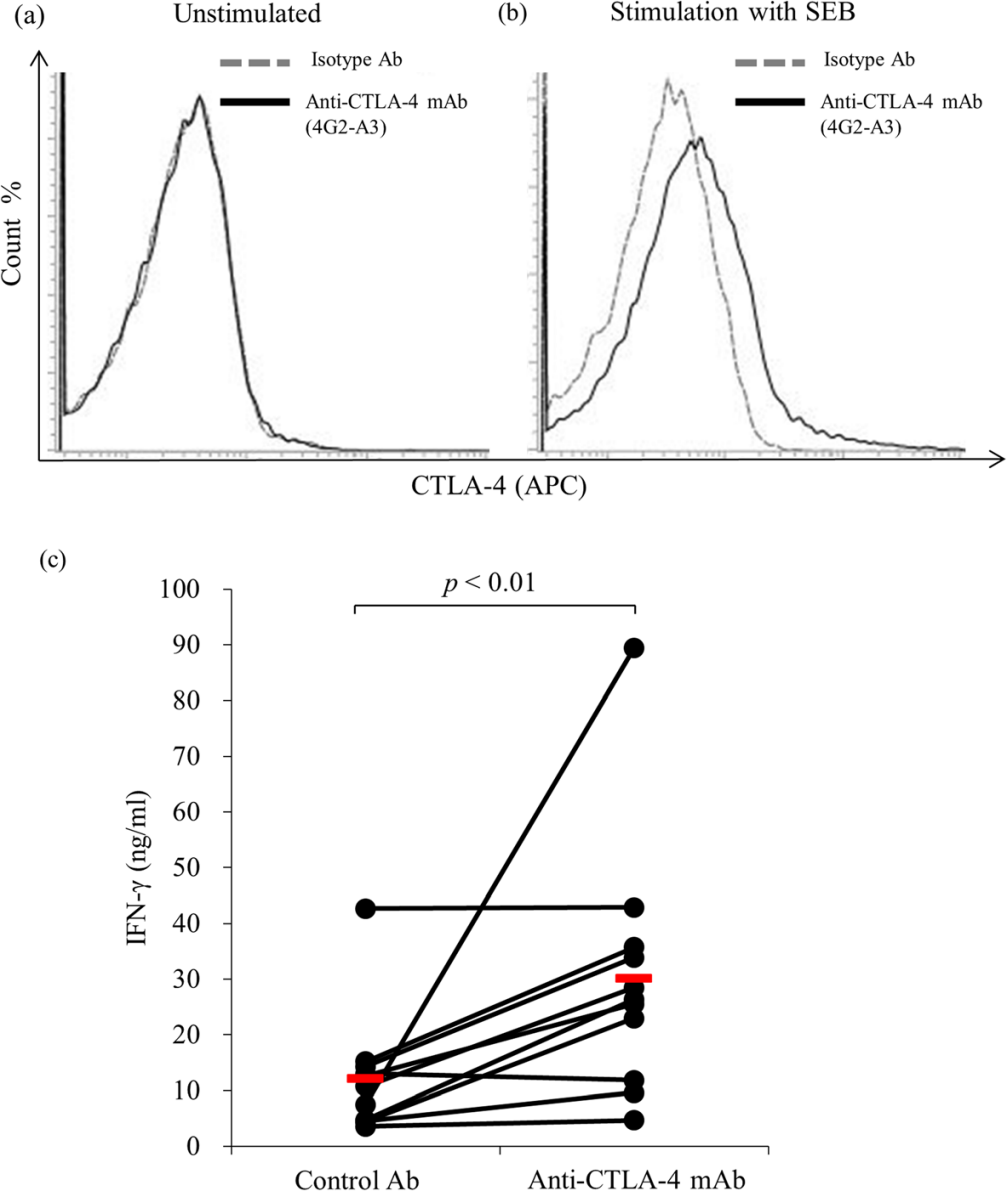
The immune activating function of established anti-CTLA-4 mAb. PBMCs were isolated from healthy cattle and cultured for 3 days in the **a** presence or **b** absence of SEB. The cultured PBMCs were harvested, and the expression of CTLA-4 was measured using flow cytometry. The lymphocyte population was gated by forward and side scattering, and the incorporation of CTLA-4 on IgM^−^ cells was measured using flow cytometry. **c** PBMCs (*n* = 11) were isolated from healthy cattle and cultured with 20 μg/ml control Ig or anti-CTLA-4 mAb in the presence of SEB. The supernatant from the culture medium was harvested after 7 days, and an ELISA was used to measure the IFN-γ concentration. The bar indicates the average of each group. Statistical comparisons between each group were made using the Wilcoxon matched-pairs test. Differences were considered statistically significant at *p* < 0.01

**Fig. 4:**
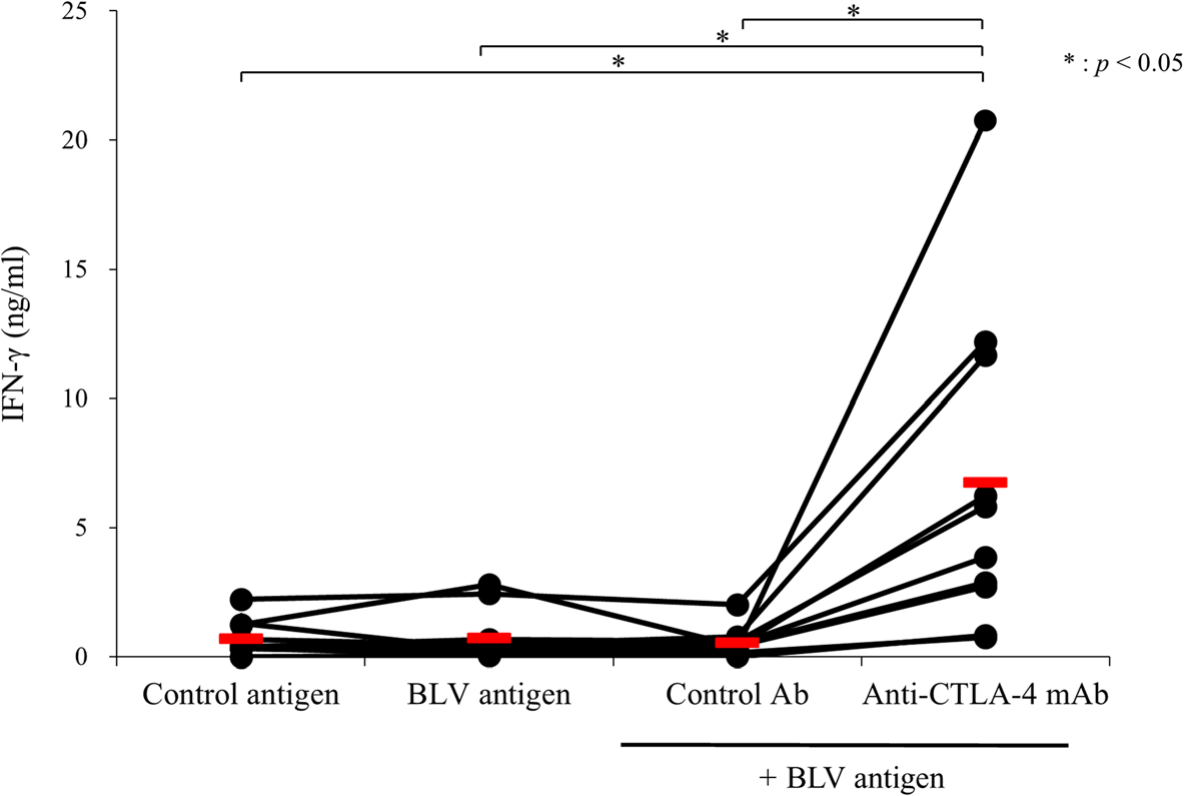
The immune activating effect of anti-CTLA-4 mAb against bovine leukemia virus (BLV) infected cattle PBMCs. PBMCs (*n* = 10) of BLV-infected cattle were cultured with 20 μg/ml control antibody or anti-CTLA-4 mAb in the presence of fetal lamb kidney cells (FLK)-BLV antigen for 7 days. FLK was used as a control antigen. The IFN-γ production was measured using an ELISA. The bars indicate the average of each group. Statistical comparisons were made using the Steel-Dwass test. The differences were considered statistically significant at *p* < 0.05

Next, we confirmed the immune activation via the CTLA-4 blockade. Bovine PBMC were isolated from healthy cattle and cultured in the presence or absence of SEB for 3 days. The cultivated PBMCs were harvested and CTLA-4 expression was measured using flow cytometry, which showed that CTLA-4 was upregulated on the activated T cells (Fig. 3a, b). Then, we added the CTLA-4 mAb into the cultivation to confirm the effect of CTLA-4 blockade in the activated cells. Interestingly, the bovine CTLA-4 mAb significantly enhanced the IFN-γ production from the activated PBMCs, while the control antibody did not (Fig. 3c). Finally, we confirmed whether CTLA-4 mAb could restore the anti-viral response by using a BLV model, since we previously showed an upregulation of CTLA-4 expression in cattle naturally infected with BLV. PBMCs were isolated from BLV-infected cattle and cultured with CTLA-4 mAb or mouse IgG isotype in the presence of BLV antigen. Interestingly, CTLA-4 blockade by the antibody significantly restored the production of IFN-γ compared with the mouse IgG isotype control (Fig. 4).

## Discussion

Several studies about immune checkpoint molecules have been conducted in humans and mice and revealed that the upregulation of molecules inhibits the host immunity, resulting in the disease progression of chronic infections and malignant neoplasms. CTLA-4 is one of the immunosuppressive molecules and its expression is reported to be upregulated in chronic infections and tumors, such as HIV, HCV, esophageal cancer, and melanoma [5, 7, 8]. In humans, the anti-CTLA-4 antibody (Ipilimumab) has already been manufactured and used on patients with melanoma. A previous study showed that the single use of ipilimumab improved the survival rate of patients with metastatic melanoma [17].

Although several studies have been performed and the therapeutic effect of anti-CTLA-4 antibody immune therapy has been reported in humans and mice, there are still limited studies concerning the function of CTLA-4 and effects of CTLA-4 blockade in cattle. In order to deepen the understanding of the mechanism of the immune response in bovine diseases, we established a bovine recombinant CTLA-4 and monoclonal antibody against bovine CTLA-4 for functional analysis. We also confirmed the immune activating effect of the bovine CTLA-4 monoclonal antibody in BLV-infected cattle.

In humans, the immune inhibitory function of CTLA-4 has been well documented and its recombinant protein has already been applied as an anti-inflammatory agent. Abatacept, a recombinant protein that combines the extracellular region of CTLA-4 and human IgG, has been applied as a biopharmaceutical for treating arthritis and juvenile idiopathic arthritis. Abatacept has been reported to reduce joint pain in arthritis patients for 104 weeks [18]. The affinity of CTLA-4 to its ligands, CD80 and CD86, is more than ten times higher than that of CD28, T cell co-stimulatory protein, and another receptor for CD80 and CD86 [19, 20]. Abatacept competitively binds to CD80 and CD86 and prevents the co-stimulation signaling from CD28 by disturbing the binding of CD28 to CD80 and CD86 for T cell activation and IFN-γ production.

In this study, bovine recombinant CTLA-4, bovine CTLA-4-Ig, was shown to bind with both bovine CD80 and CD86, and bovine CTLA-4-Ig significantly inhibited IFN-γ production from activated PBMCs. These findings suggest that bovine CTLA-4 has an immune inhibitory function like in humans, and thus could be used as an anti-inflammatory drug in cattle. Further investigation is needed to verify this possibility.

In a previous report, we indicated that the expression of CTLA-4 was closely associated with disease progression relating to immunosuppression in BLV-infected cattle [16]. This observation indicates that CTLA-4 could be related to immunosuppression during BLV infection. In contrast, blockade of the CTLA-4 pathway may show the potential for the development of new therapies against chronic infectious diseases and cancers.

Ipilimumab, a humanized monoclonal antibody against CTLA-4, has been applied as a biopharmaceutical for treating melanoma in humans. In addition, it is undergoing clinical trials for the treatment of several cancers, including non-small cell lung carcinoma, small cell lung cancer, bladder cancer, and metastatic hormone-refractory prostate cancer. In this study, we established an anti-bovine CTLA-4 antibody to confirm the immune enhancing effect. An established CTLA-4 mAb (4G2-A3) exhibited specific binding against bovine CTLA-4, not CD28, which is homologous to CTLA-4. Fortunately, the bovine CTLA-4 mAb significantly restored the IFN-γ production from the PBMCs treated by inhibitory CTLA-4-Ig and CTLA-4 upregulating PBMCs by disturbing the binding of CTLA-4 to CD80 or CD86. Furthermore, the CTLA-4 blockade by the antibody enhanced IFN-γ production from the BLV-infected cattle-derived PBMCs stimulated with BLV-antigens. These results indicate that CTLA-4 mediates the functional exhaustion of CD4^+^ and CD8^+^ T cells and is associated with disease progression in BLV-infected cattle.

Several combination therapies have been confirmed to enhance immune therapy in humans and mice. Recently, the administration of a combination of the anti-CTLA-4 antibody and anti-PD-1 antibody is known to be the most promising combination therapy. It has been reported that 35 (74%) out of 47 patients had disease progression or died with anti-CTLA-4 antibody therapy alone. On the other hand, only 43 (45%) out of 93 patients had disease progression or died with the combination therapy of the anti-CTLA-4 antibody and anti-PD-1 antibody [21].

Finally, in order to elucidate the synergistic effect of the combination therapy in cattle, we co-cultured bovine PBMC from BLV-infected cattle with the anti-CTLA-4 and anti-PD-L1 antibodies. Although the IFN-γ production was increased by anti-CTLA-4 or PD-L1 antibodies, we did not observe a synergistic effect, as has been reported in humans and mice (data not shown). This might be because the established antibody’s activating effect was not enough to induce a synergistic effect. Thus, establishment of a new anti-CTLA-4 antibody might be needed to construct the combination therapy.

## Conclusions

In our study, we observed the immune inhibitory function of bovine CTLA-4 by using recombinant bovine CTLA-4-Ig. We immunized recombinant bovine CTLA-4-Ig to mice and established anti-bovine CTLA-4 antibody. For the established antibody, we confirmed the binding, blocking and immune activating abilities. The established antibody specifically bound to CTLA-4 and blocked the binding of CTLA-4 with CD80/CD86. Anti-CTLA-4 antibody significantly increased the IFN-γ production from PBMCs of healthy and BLV infected cattle in vitro. These results suggest that anti-CTLA-4 antibody has a potential to develop a novel therapy against BLV infection. However, we did not observe a synergistic effect with the co-administration of the PD-L1 antibody. In order to activate immune response more, we will investigate the synergistic effects of several combinations with other CTLA-4 mAb clones.

## Methods

### Samples from cattle

The blood samples of healthy cattle were collected from dairy cattle kept in Hokkaido University, and those of BLV infected cattle were collected from dairy farms in Hokkaido, Japan between 2014 and 2016. Informed consent was fully obtained from the farmers. These blood samples were analyzed within 2 days.

### Generation of recombinant bovine CTLA-4, CD80 and CD86 immunoglobulin fusion protein

The primers were designed based on bovine CTLA-4 (NM_174297), bovine CD80 (NM_001206439), and bovine CD86 (NM_001038017) to amplify the extracellular region fragment of each molecule (Table 1). After the extracellular region fragments were amplified, they were inserted into the cloning site of a modified pCXN2.1-Rabbit IgG Fc vector (provided by Dr. Yokomizo, Juntendo University, Japan). The complete expression vectors, pCXN2.1-Rabbit IgG Fc-bovine CTLA-4, pCXN2.1-Rabbit IgG Fc-bovine CD80, and pCXN2.1-Rabbit IgG Fc-bovine CD86 were transfected into Expi293F cells (Thermo Fisher Scientific, Waltham, MA, USA) by ExpiFectamine (Thermo Fisher Scientific) and purified from culture medium harvested on day two by Ab-Capcher ExTra (ProteNova, Kagawa, Japan) according to the manufacturer’s protocol. After purification, the buffer solution was replaced by PBS pH 7.2 (FUJIFILM Wako Pure Chemical, Osaka, Japan) using a PD midiTrap G-25 (GE Healthcare, Little Chalfont, UK) according to the manufacturer’s protocol. The expression and purification of CTLA-4-Ig, CD80-Ig, and CD86-Ig were confirmed by SDS-PAGE. The concentrations of CTLA-4-Ig, CD80-Ig, and CD86-Ig were confirmed using a rabbit IgG ELISA Quantitation Set (Bethyl Laboratories, Montgomery, TX, USA) and Pierce BCA Protein Assay Kit (Thermo Fisher Scientific) according to the manufacturer’s protocol.

**Table 1:** The list of primer used in the experiment

Primer	Primer sequences (5'-3')	Restriction enzyme
BLV diagnosis		
BLV-LTR-1	TGTATGAAAGATCATGCCGAC	
BLV-LTR-533	AATTGTTTGCCGGTCTCT	
BLV-LTR-256	GAGCTCTCTTGCTCCCGAGAC	
BLV-LTR-453	GAAACAAACGCGGGTGCAAGCCAG	
β-globin Forward (F)	ACACAACTGTGTTCACTAGC	
β-globin Reverse (R )	CAACTTCATCCACGTTCACC	
Establishment of expression vector		
pCXN2.1-CTLA-4-Ig F	CGCGGATATCATGGCTTGCTCTGGATTCCA	*EcoR*V
pCXN2.1-CTLA-4-Ig R	CGGGGTACCATCAGAATCCGGGCATGGTT	*Kpn*I-HF
pCXN2.1-CD80-Ig F	CGCGGATATCATGGGTCACACAATGAAGTG	*EcoR*V
pCXN2.1-CD80-Ig R	CGGGGTACCGGTCCAGGTCAGGTGCTGAT	*Kpn*I-HF
pCXN2.1-CD86-Ig F	CGCGGATATCATGCGTTTCAAATGCACCAT	*EcoR*V
pCXN2.1-CD86-Ig R	CGGGGTACCTGGGACAGGGGGGCTTGGCA	*Kpn*I-HF
pEGFP-N2-CTLA-4 F	GGAAGATCTATGGCTTGCTCTGGATTCCA	*Bgl*II
pEGFP-N2-CTLA-4 R	CCGGAATTCATTGATGGGAATAAAATAAG	*EcoR*I
pEGFP-N2-CD28 F	GGAAGATCTATGCTCAGGCTGCTCCTGGC	*Bgl*II
pEGFP-N2-CD28 R	TCCCCCGGGGGAGCGGTAGGCCGCAAAGT	*Sma*I

^*^Recognition sites of restriction enzymes were underlined

### Generation of bovine CTLA-4, CD80, CD86, and CD28 expressing cells

In order to construct EGFP fusion expression vectors of bovine CTLA-4, CD80, CD86, and CD28, we designed the primers to amplify the ORF region that did not have a stop codon. The amplified region fragment of bovine CTLA-4, CD80, CD86, and CD28 was inserted into the cloning site of a pEGFP-N2 vector (Clontech, Palo Alto, CA, USA). The complete vectors, pEGFP-N2-CD80, pEGFP-N2-CD86, and pEGFP-N2-CD28 were transfected into Cos-7 cells by Lipofectamine 2000 reagent (Thermo Fisher Scientific) and cultured for 48 h. The complete vector, pEGFP-N2-CTLA-4 was transfected into CHO-DG44 cells (provided by Dr. Suzuki, Hokkaido University, Japan) with the Lipofectamine LTX reagent (Thermo Fisher Scientific). CTLA-4-EGFP expressing CHO-DG44 cells were cloned and established CTLA-4-EGFP highly expressing CHO-DG44 cells. The binding of CTLA-4-Ig to the CD80 or CD86 expressing cells or the binding of CD80-Ig or CD86-Ig to CTLA-4 expressing cells was confirmed using flow cytometry with FACSVerse (BD Biosciences, San Jose, CA, USA) and FCS Express 4 (De Novo Software, Glendale, CA, USA) as previously described with some modifications [22]. Rabbit IgG (Southern Biotech, Birmingham, AL, USA) was used as a control Ig.

### Functional analysis of CTLA-4-Ig

PBMC (1 × 10^6^ cells/0.2 ml) were isolated from healthy cattle (maintained at the Field Science Center for Northern Biosphere, Hokkaido University) as previously described, and cultured with 10 nM of CTLA-4-Ig or rabbit IgG (Southern Biotech) in the presence of 0.1 μg/ml Staphylococcal enterotoxin B from *Staphylococcus aureus* (SEB) (Sigma-Aldrich, St. Louis, MO, USA) to determine the inhibitory effect of CTLA-4-Ig. After 7 days, the culture medium was harvested and an ELISA was used to measure the IFN-γ concentration for bovine IFN-γ (Mabtech, Nacka Strand, Sweden) according to the manufacturer’s protocol.

### Establishment of bovine CTLA-4 specific mAb

Establishment of anti-bovine CTLA-4 mouse mAb was outsourced to Cell Engineering Corporation (Osaka, Japan). The reactivity of polyclonal antibodies from hybridomas was screened using ELISA. Bovine CTLA-4 monoclonal antibody was selected by flow cytometry using CTLA-4-EGFP expressing CHO-DG44 cells as described above. An anti-bovine CTLA-4 monoclonal antibody (4G2-A3) was selected by the screening and purified for this study. The specificity of the monoclonal antibody to CTLA-4 was confirmed using flow cytometry. In brief, CTLA-4 or CD28 expressing cells were incubated in PBS containing 10% goat serum (Sigma-Aldrich) at room temperature for 15 min to suppress nonspecific binding to the Fc receptor. After pretreatment, the cells were incubated with 10 μg/ml anti-CTLA-4 mAb or a control Ab (mouse IgG_1_, Southern Biotech) for 20 min at room temperature. The cells were washed twice, and anti-CTLA-4 mAb was detected with Alexa Fluor 647-conjugated anti-mouse IgG (H + L) F (ab’)_2_ (Thermo Fisher Scientific).

### Confirmation of the blocking ability of the bovine CTLA-4 specific mAb (4G2-A3)

The blocking ability of the anti-CTLA-4 mAb was confirmed using flow cytometry with CD80-Ig or CD86-Ig and CTLA-4-EGFP expressing CHO-DG44 cells. CTLA-4 expressing cells were incubated in PBS containing 10% goat serum^12^ at room temperature for 15 min. After pretreatment, the cells were incubated with different concentrations of anti-CTLA-4 mAb (1.25, 2.5, 5.0, 10, and 20 μg/ml) for 20 min at 25 °C. Mouse IgG_1_ (Southern Biotech) was used as an isotype antibody. The cells were washed twice, and 0.2 μg/ml CD80-Ig or CD86-Ig was then added. After incubating for 20 min at 25 °C, the cells were washed twice. CD80-Ig or CD86-Ig was detected using flow cytometry with Alexa Fluor 647-conjugated anti-rabbit IgG (H + L) goat IgG (Thermo Fisher Scientific).

### Blocking assay with the anti-bovine CTLA-4 antibody

Firstly, we confirmed the direct effect by the addition of the anti-bovine CTLA-4 antibody in an immune inhibitory assay using CTLA-4-Ig as mentioned above. Briefly, PBMCs were cultured with 10 nM CTLA-4-Ig or rabbit IgG (Southern Biotech) in the presence of 0.1 μg/ml SEB (Sigma-Aldrich), and then 20 μg/ml anti-bovine CTLA-4 antibody or control Ab (mouse IgG, Sigma-Aldrich) was added. After 7 days, the culture medium was harvested and an ELISA was used to measure the IFN-γ concentration. We also confirmed the effects of the antibody in cells that highly express CTLA-4. To increase the expression of CTLA-4 in PBMCs, they were cultivated for 3 days at 37 °C in the presence or absence of 0.1 μg/ml SEB (Sigma-Aldrich). The increase of CTLA-4 expression was confirmed using flow cytometry. Briefly, harvested cells were stained with anti-CTLA-4 mAb (4G2-A3) or mouse IgG_1_ (Southern Biotech) for 20 min at 37 °C. After washing, cells were stained with Alexa Fluor 647-conjugated anti-mouse IgG (H + L) F (ab’)_2_ (Thermo Fisher Scientific) for 20 min at room temperature. Next, we stained the cells with PE/Cy7 conjugated anti-IgM antibody (Bio-Rad, Hercules, CA, USA) for 15 min at room temperature. The anti-IgM antibody was conjugated by using Lightning-Link Conjugation Kits (Innova Biosciences, Cambridge, UK) according to the manufacturer’s protocol. Then, cells were washed and analyzed using FACS Verse (BD Biosciences) and FCS Express 4 (De Novo Software). After the confirmation of a high CTLA-4 expression, the PBMCs were cultured with 20 μg/ml anti-bovine CTLA-4 antibody or mouse IgG (Sigma-Aldrich) with 0.1 μg/ml SEB (Sigma-Aldrich) for 7 days. The culture medium was harvested, and an ELISA was used to measure the IFN-γ concentration.

Finally, we confirmed the immune activating function of the anti-CTLA-4 antibody in PBMCs of BLV-infected cattle (maintained at dairy farms in Hokkaido). The BLV diagnosis and PBMCs blockade assay were conducted as previously described with some modifications [23]. Briefly, PBMCs derived from BLV-infected cattle were cultured with 20 μg/ml anti-bovine CTLA-4 antibody or mouse IgG (Sigma-Aldrich) in the presence of heat-inactivated supernatant (2%) from BLV-infected fetal lamb kidney cells (FLK-BLV) for 7 days at 37 °C in 5% CO_2_. The FLK-BLV supernatant contained BLV-antigens to stimulate BLV-specific T cells as previously described with some modifications [24]. The heat-inactivated supernatant (2%) of BLV-uninfected FLK was used as a negative control antigen. The culture medium was harvested after 7 days, and an ELISA was used to measure the IFN-γ concentration. Furthermore, we evaluated whether the anti-bovine CTLA-4 antibody enhances PD-L1-induced IFN-γ production. PBMCs derived from BLV-infected cattle were cultured with 10 μg/ml anti-bovine CTLA-4 antibody and anti-bovine PD-L1 antibody (4G12, rat IgG2a) with FLK-BLV for 7 days at 37 °C in 5% CO_2_. Mouse IgG (Sigma-Aldrich) and rat IgG (Sigma-Aldrich) were used as control antibodies of the anti-CTLA-4 antibody and anti-bovine PD-L1 antibody, respectively. The culture medium was harvested, and an ELISA was used to measure the IFN-γ concentration.

### Statistics

The Wilcoxon rank sum test was used for a two-group comparison, and Steel-Dwass test was used for comparisons when there were more than three groups. *P*-values of < 0.01 or < 0.05 were considered statistically significant.

## Data Availability

The datasets used and/or analyzed during the current study are available from the corresponding author on reasonable request.

## Abbreviations

AL: Aleukemic leukemic
BLV: Bovine leukemia virus
Con A: Concanavalin A
CTLA-4: Cytotoxic T-Lymphocyte Antigen-4
EBL: Enzootic bovine leukemia
EGFP: Enhanced green fluorescent protein
ELISA: Enzyme-Linked Immunosorbent Assay
FLK: Fetal Lamb Kidney cells
HCV: Hepatitis C Virus
HIV: Human immunodeficiency virus
IFN-γ: Interferon gamma
LAG-3: Lymphocyte Activation Gene three
mAb: Monoclonal antibody
NK cell: Natural Killer cell
ORF: Open Reading Frame
pAb: Polyclonal antibody
PBMC: Peripheral blood mononuclear cell
PBS: Phosphate Buffered Saline
PD-1: Programmed cell death-one
PD-L1: Programmed cell death ligand-one
PL: Persistent Lymphocytosis
SDS-PAGE: Sodium Dodecyl Sulfate-polyacrylamide Gel Elactrophoresis
SEB: Staphlococcal Enterotoxin B
TGF-β: Transforming growth factor beta
TNF-α: Tumor necrosis factor alpha
